# Sleep problems from age two to five years and neurological outcome in children born extremely preterm: a cross-sectional study

**DOI:** 10.3389/fped.2025.1562630

**Published:** 2025-07-09

**Authors:** Kristine Marie Stangenes, Mari Hysing, Maria Vollsæter, Irene Bircow Elgen, Trond Markestad, Bjørn Bjorvatn

**Affiliations:** ^1^ Department of Global Public Health and Primary Care, University of Bergen, Bergen, Norway; ^2^ Department of Health Registry Research and Development, Norwegian Institute of Public Health, Bergen, Norway; ^3^ Department of Psychosocial Science, Faculty of Psychology, University of Bergen, Bergen, Norway; ^4^ Department of Clinical Science, University of Bergen, Bergen, Norway; ^5^ Department of Child and Adolescent Mental Health Services, Haukeland University Hospital, Bergen, Norway; ^6^ Norwegian Competence Center for Sleep Disorders, Haukeland University Hospital, Bergen, Norway

**Keywords:** sleep, sleep problems, extremely preterm birth, neurodevelopment, cognition, minor motor problems

## Abstract

**Introduction:**

Premature birth is associated with a higher risk of sleep problems and neurodevelopmental disabilities (NDD). We examined relationships between sleep problems and cognitive, motor, and sensory functions in a national cohort of five-year-old children born extremely preterm (EPT) with the purpose of identifying possible means of improving developmental outcomes.

**Methods:**

This study was part of a national cohort study of all children born extremely preterm, defined here as gestational age less than 28 completed weeks, or birth weight below 1,000 g, born in Norway in 1999 and 2000. Parents completed a structured retrospective questionnaire at age five to assess sleep problems from ages two to five years. We assessed cognitive function using the Wechsler Preschool and Primary Scale of Intelligence-Revised (WPPSI-R), evaluated motor function with the Movement Assessment Battery for Children (M-ABC) and classified cerebral palsy (CP) according to the Gross Motor Function Classification System (GMFCS). NDD was graded from no NDD (no disabilities) to NDD 2 and 3 (moderate and severe disability).

**Results:**

Of 372 eligible children, 253 (68%) participated. Parents reported that 28.5% had general sleep problems from ages two to five years. Prevalences of specific problems were 21.7% for nighttime awakenings, 17.8% for difficulty falling asleep, 5.9% for early morning awakening and 1.6% for late morning awakening. Children with Full scale IQ < 85 were at increased risk of general sleep problems (adjusted odds ratio - aOR 1.8), as well as nighttime awakenings (aOR 2.8), and early morning awakenings (OR 2.9), but not for difficulty falling asleep compared to those with higher IQ levels.

EPT children with moderate to severe NDD (NDD 2 and 3) showed a higher prevalence of general sleep problems [adjusted odds ratio (aOR) 3.9], nighttime awakenings (aOR 4.8), and early morning awakenings (OR 7.9) compared to those with no NDD (NDD 0).

**Conclusion:**

General and specific sleep problems were associated with low cognitive function and moderate to severe NDD. Our findings underscore the importance of addressing sleep within a comprehensive care framework for EPT children and highlight the need for addressing and target interventions for sleep problems.

## Introduction

Preterm birth can disrupt normal brain development and cause a syndrome described as “encephalopathy of prematurity” (1). The outcome may be various degrees of neurodevelopmental disabilities (NDD), i.e., cognitive, behavioral, motor, and sensory disorders, of which cognitive difficulties are the most common sequelae (2). The risk of NDD increases with decreasing gestational age (GA) at birth (3–5). Emerging evidence also suggests that prematurity is associated with an increased risk of sleep problems during childhood (6–8), including the crucial preschool years (9, 10). Additionally, it may lead to disrupted circadian rhythm, for instance causing early morning awakening (11–13) during childhood.

In addition, sleep itself plays a pivotal role in brain maturation during early childhood (14), and disrupted sleep may adversely affect various developmental outcomes, including cognitive function (15). Children with various neurological disorders, including cerebral palsy (16) and low cognitive function (17–19), often experience sleep problems, but to what extent sleep disorders contribute to the severity of NDD is uncertain and may vary.

Preterm-born children may be more susceptible to the negative impact of disturbed sleep on cognitive function than term-born children (20). Prematurity affects both sleep architecture (21) and neurodevelopment (3), with research showing that specific electroencephalographic (EEG) sleep measures correlate with lower neurodevelopmental performance in extremely preterm children at age 2 (22) and in healthy very preterm children at ages 12–24 months (23).

However, the intricate nature of these relationships remains poorly understood (24), and there is limited research on the connection between neurological sequelae and sleep problems in children born preterm. Studies have suggested that reduced sleep efficiency (20), reduced sleep quality (25), and increased variation in sleep patterns (26, 27) are associated with impaired cognitive function in prematurely born children. However, these studies addressed other sleep parameters and more mature premature infants than those in our study.

In a Norwegian national follow-up study of children born extremely preterm (EPT), we have previously found that the prevalence of sleep problems at 11 years of age increased from 20% among children with no disability (NDD 0) to 67% among children with severe disability (NDD 3) (28). The current study is based on the same cohort and explores the prevalence of general and specific sleep problems in EPT children aged two to five years and their association with cognitive and motor difficulties and severity of NDD.

Understanding the prevalence and implications of sleep problems in young EPT children is crucial, as early interventions could correlate with long-term cognitive and developmental challenges. Therefore, this study seeks to answer the following research question: What is the prevalence of general and specific sleep problems in EPT children aged two to five years, and how are these issues related to cognitive and motor difficulties, as well as the severity of neurodevelopmental disabilities? We hypothesize that EPT children will exhibit a high prevalence of sleep problems, and that these sleep problems will be significantly associated with greater severity of neurological outcome by age five.

## Materials and methods

### Population

This study was part of a national cohort study of all children born EPT, defined here as GA less than 28 completed weeks, or birth weight (BW) below 1,000 g, born in Norway in 1999 and 2000 (29, 30). We defined children as born small for gestational age (SGA) if the BW was below the 10th percentile for GA and sex for Norwegian children (31). The children were prospectively followed from birth. At five years of age, the parents completed questionnaires addressing the child's sleep behavior, physical and mental health, and social circumstances. These questionnaires were sent to them by mail around the same time as the children were examined at the 19 participating pediatric departments in accordance with the research protocol (32).

### Sleep questions

The parents were asked if the child had experienced sleep problems after the age of two years. The response options were: “no sleep problems during these years”, “previous sleep problems but not within the last year” and “still experiencing sleep problems”. A child who either had “previous sleep problems but not within the last year” or “still experiencing sleep problems” was defined as having “general sleep problems between the ages of two and five”. For these children, the parents were asked to specify the nature of the problems by ticking the applicable options: “difficulty falling asleep at night”, “waking up during the night”, “waking up unusually early”, “waking up unusually late” or “other kinds of sleep problems”.

### Clinical assessments

Experienced pediatricians conducted general clinical and neurological examinations. Visual and auditory functions were assessed during the clinical examination or were based on records from prior assessments at public healthcare clinics.

Physiotherapists assessed motor functions using the Movement Assessment Battery for Children (M-ABC) test (33). The M-ABC test evaluates motor function in children through eight tasks, which address hand motor skills, ball skills, and balance. Scoring for each task ranges from 0 to 5, where higher scores denote poorer motor performance. A cumulative score above the 95th percentile of age-specific reference values is indicative of a motor skill problem according to the test manual (33).

The M-ABC is categorized into different age bands; our study employed age band 1, which covers four to six year-old children (33).

Motor skills of children with cerebral palsy (CP) were categorized according to *the Gross Motor Function Classification System (GMFCS)* (34). The GMFCS is a five-level classification: Class 1, means that the child can move freely around in home, in school with more; Class 2, unable to run or jump; Class 3, dependent on devices for walking; and Classes 4 and 5, non-ambulatory CP (34).

Psychologists evaluated cognitive abilities using the Wechsler Preschool and Primary Scale of Intelligence-Revised (WPPSI-R) (35). WPPSI-R is tailored for children between two years and 11 months and seven years and three months. It comprises 12 subtests, which are divided into two categories: six assess verbal IQ, and six performance IQ. A Full Scale Intelligence Quotient (FIQ) is derived from these subscales (35). The reference mean value for the FIQ scores is 100, and the standard deviation (SD) is 15. A borderline FIQ is defined as 1–2 SDs below the mean, i.e., 70–84.

Based on these assessments, each child was assigned a Neurodevelopmental Disability (NDD) score according to the classifications defined in the Extremely Preterm Infants (surfactant C) cure (EPIcure) study, i.e., no, minor, moderate or severe disability (32, 36).

No NDD (No identified disability) was defined as normal vision and hearing, no cerebral palsy, an M-ABC score at or below the 95th percentile, and a FIQ of 85 or higher. NDD 1 (minor disability) included one or more of the following: squint/refractive error, mild hearing loss, cerebral palsy class 1, M-ABC score above the 95th percentile, or FIQ between 1 and 2 SDs below the reference mean value (i.e., 70–84). NDD 2 (moderate disability) included severe visual impairment, need of hearing aid, cerebral palsy class 2–3, or FIQ between 2 and 3 SDs below the reference mean value (i.e., 55–70). NDD 3 (severe disability) included legal blindness, complete deafness, cerebral palsy class 4–5, or FIQ more than 3 SDs below the reference mean value of 100 (i.e., <55).

### Statistical analyses

To evaluate sample representativeness, we compared mean GA, mean BW, proportion of SGA children, and prevalence of periventricular haemorrhage grade three or four between participants and non-participants. We used *t*-tests for continuous variables and chi-square tests for categorical variables for comparisons between participants and non-participants, as well as for comparing the prevalence of various sleep problems among EPT children with and without CP, and among EPT children with and without SGA status at birth. The significance level was set at an α-level of 0.05.

We used logistic regression analyses to assess the risks, expressed as odds ratios (OR) with 95% confidence intervals (CI), of the various sleep problems according to M-ABC score (dichotomized as normal and abnormal, defined as a score at or above the 95th percentile or lower), FIQ score (dichotomized as normal and score below 85), and NDD groups both unadjusted and after adjusting for SGA vs. non-SGA birth and maternal education (dichotomized as at least a three-year college education or less).

We adjusted for the following parameters: low socioeconomic status and intrauterine growth restriction (born SGA), as both are associated with preterm birth (37), sleep problems (38, 39), and a child's FIQ (40, 41). Notably, the relationship between SGA and a child's FIQ primarily applies to children born prematurely (41). Our previous study also identified SGA as a risk factor for sleep problems in children born prematurely (42). We also performed the same regression analyses with M-ABC and FIQ scores as continuous variables.

### Ethics

The study was approved by the Regional Committees for Medical and Health Research Ethics (REC numbers: 2009/2271, 2017/1174) and the Norwegian Data Protection Authority. Informed written consent was obtained from the parents.

## Results

### Study population

There were no significant differences in mean GA, mean BW, proportion of SGA children, or prevalence of neonatal periventricular hemorrhage grade three or four between participants (*n* = 253) and non-participants (*n* = 119) (data not shown).

**Figure 1:**
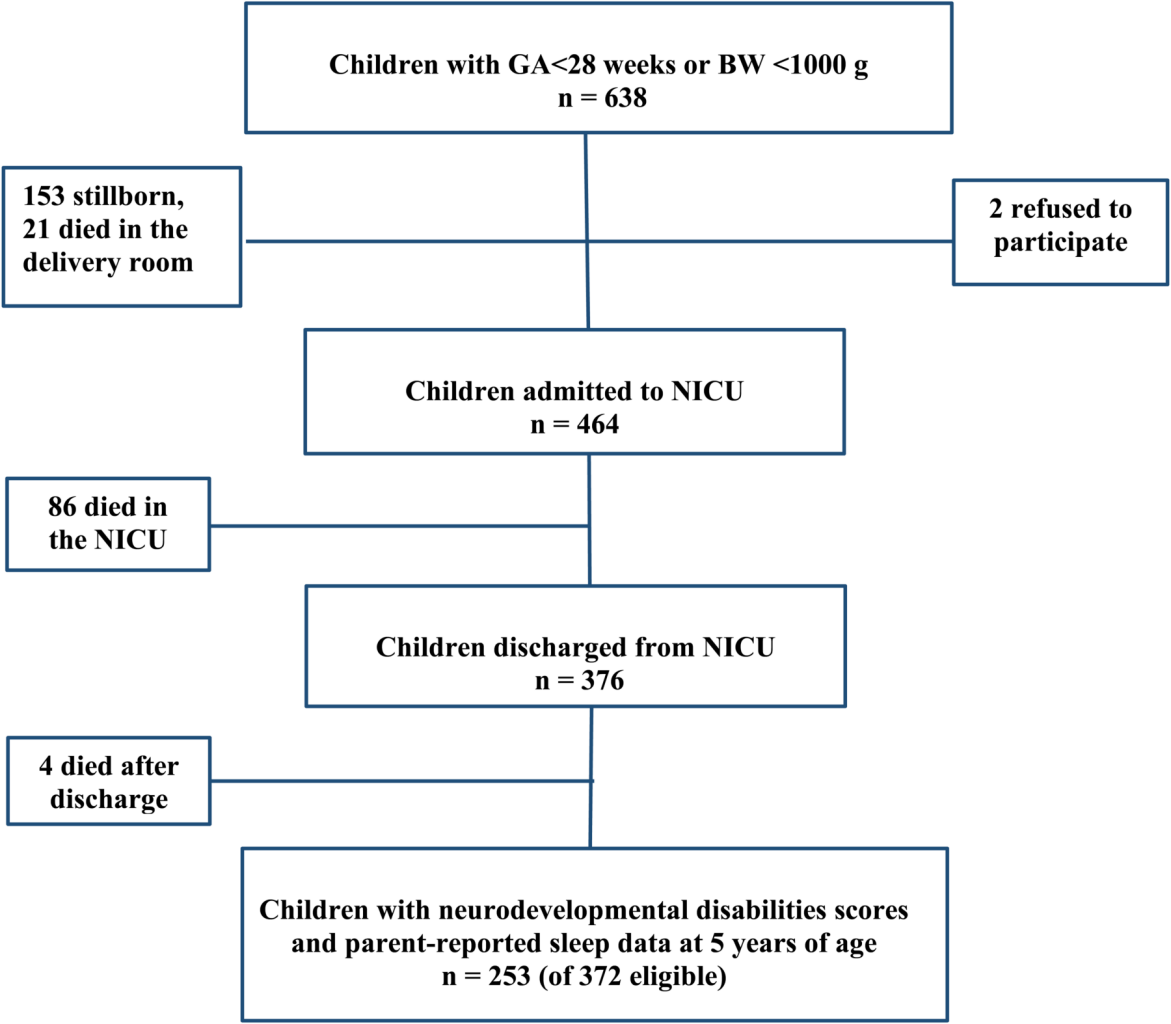
The recruitment of participants in a follow-up study of a national cohort of children born extremely preterm^a^ in Norway, 1999–2000. ^a^Gestational age <28 weeks or birth weight <1,000 g. BW, birthweight, GA, gestational age, NICU, neonatal intensive care unit.

Sleep and NDD data were obtained for 253 of the 372 (68%) EPT children who were alive at five years of age (Figure 1). Parent and child characteristics are described in Table 1. FIQ scores were available 244 (96%) and M-ABC scores for 230 (91%) of the children. The mean FIQ score was 93.2 (range 48–130) for all, and 94.2 (range 53–130, *n* = 229) when children with CP were excluded. Mean M-ABC score was 9.8 (range 0–38) for all, and 9.6 (range 0–38, *n* = 224) when children with CP were excluded. Of the 253 children, 117 (46%) had no NDD (NDD 0), 100 (40%) had minor (NDD 1), 23 (9%) moderate (NDD 2), and 13 (5%) severe disabilities (NDD 3).

**Table 1:** Characteristics of the 253 children born extremely preterm^a^ in Norway, 1999–2000, with neurodevelopmental disability (NDD) score and parent-reported sleep data at five years of age.

Characteristics	Mean (range)
Gestational age, weeks	26.4 (23–31)
Birth weight, grams	849 (450–1370)
	% (*n*)
Singeltons	76.3 (193)
Boy	56.5 (143)
Small for gestational age	18.6 (47)
Periventricular hemorrhage grade 3 or 4	9.1 (23)
Assessment at 5 years of age	Mean (range)
FIQ^b^ (*n* = 244)	93.2 (48–130)
Total M-ABC^d^ test score (*n* = 230)	9.84 (0,38)
	% (*n*)
FIQ^b^ <85	26.1 (66)
M-ABC^c^ >95	15.4 (39)
Cerebral palsy at 5 years of age	8.7 (22)
Epilepsy at 5 years of age	5 (14)
Use of asthma medications after 2 years of age	35.2 (89)
Higher education mother^d^	45.5 (115)
Current smoking mother	26.1 (66)
Current smoking father	24.9 (63)

^a^ Gestational age <28 weeks or birth weight <1,000 g.

^b^ Full IQ.

^c^ Movement Assessment Battery for Children.

^d^ Defined as 3-year college education or more.

### Sleep problems

General sleep problems were reported for 28.5% of the children. Nighttime awakenings were the most common problem (21.7%) followed by difficulty falling asleep (17.8%) (Table 2). Only four children were reported to wake up unusually late. Therefore, this sleep variable was not included in further analyses. Additionally, 6.7% (*n* = 17) were reported to have other sleep problems, which were not included in further analyses (Table 2). The prevalence of the various sleep problems did not differ between sexes (data not shown).

**Table 2:** Prevalence of parent-reported sleep problems at two to five years in children born extremely preterm^a^ in Norway, 1999–2000. (*n* = 253).

Prevalence of parent-reported sleep problems	% (*n*)
General sleep problems	28.5 (72)
Specific sleep problems	
Difficulty falling asleep	17.8 (45)
Awakenings during the night	21.7 (55)
Wake up unusually early	5.9 (15)
Wake up unusually late	1.6 (4)
Other sleep problems	6.7 (17)

^a^ Gestational age <28 weeks or birth weight <1,000 g.

### Sleep problems in relation to CP and SGA

Children with CP (*n* = 22) had a higher prevalence of general sleep problems (31.8% vs. 28.1%, *p* = 0.72), nighttime awakenings (31.8% vs. 20.8%, *p* = 0.23), and other sleep problems (9.1% vs. 6.5%, *p* = 0.64) compared to children without CP; however, these differences were not statistically significant. They also showed a non-significant lower prevalence of difficulty falling asleep (13.6% vs. 18.2%, *p* = 0.59) and waking up unusually early (4.5% vs. 6.1%, *p* = 0.77).

The prevalence of sleep problems was higher in SGA children (*n* = 47) compared to non-SGA children (*n* = 206), but only differences in general sleep problems (40.4% vs. 25.7%, *p* = 0.044) and nighttime awakenings (38.3% vs. 18.0%, *p* = 0.002) were statistically significant.

### Sleep problems and FIQ score

Children with FIQ of less than 85 had a significantly higher risk of general sleep problems, nighttime awakenings, and early morning awakenings compared to children with an FIQ of 85 or above. The odds ratio for nighttime awakenings remained significant after adjusting for maternal education and SGA vs. non-SGA (Table 3).

**Table 3:** Parent-reported sleep problems at two to five years in children born extremely preterm^a^ in Norway, 1999 and 2000, according to intelligence quotient (IQ) at five years. (*n* = 244).

General sleep problems (yes)	% (*n*)	Unadjusted OR (95% CI)	Adjusted^b^ OR (95% CI)
IQ ≥ 85	23.6 (42)	1.00	1.00
IQ < 85	37.9 (25)	1.97 (1.08–3.62)	1.84 (0.99–3.42)
Difficulty falling asleep (yes)	% (*n*)	Unadjusted OR (95% CI)	Adjusted^b^ OR (95% CI)
IQ ≥ 85	15.2 (27)	1.00	1.00
IQ < 85	22.7 (15)	1.65 (0.81–3.33)	1.50 (0.73–3.09)
Awakenings during the night (yes)	% (*n*)	Unadjusted OR (95% CI)	Adjusted^b^ OR (95% CI)
IQ < 85	15.7 (28)	1.00	1.00
IQ < 85	33.3 (22)	2.68 (1.39–5.14)	2.77 (1.40–5.45)
Wake up unusually early (yes)	% (*n*)	Unadjusted OR (95% CI)	Adjusted^b^ OR (95% CI)
IQ < 85	3.9 (7)	1.00	
IQ < 85	10.6 (7)	2.89 (0.976–8.61)	^c^

OR, odds ratio, CI, confidence interval.

^a^ Gestational age <28 weeks or birth weight <1,000 g.

^b^ Adjusted for SGA and maternal education (dichotomized as less than a 3-year college education or not).

^c^ Analysis not conducted due to small sample sizes.

There was also an increased risk of general sleep problems [OR 0.98, confidence interval (CI) 95% (0.96, 0.99)], nighttime awakenings [OR 0.98, CI 95% (0.96, 0.99)], and early morning awakenings [OR 0.96, CI 95% (0.93, 0.99)] associated with lower FIQ when included as a continuous variable in the regression model (data not shown).

### Sleep problems and M-ABC score

The prevalence of any sleep problem did not differ between children with M-ABC score >the 95th and those with lower scores (Table 4). Similarly, M-ABC score was not associated with sleep problems when included as a continuous variable in the regression model (data not shown).

**Table 4:** Parent-reported sleep problems at two to five years in children born extremely preterm^a^ in Norway, 1999−2000, according to M-ABC^c^ test score at five years. (*n* = 230).

General sleep problems (yes)	% (*n*)	Unadjusted OR (95% CI)	Adjusted^b^ OR (95% CI)
M-ABC test score ≤ 95th percentile	25.7 (49)	1.00	1.00
M-ABC test score > 95th percentile	35.9 (14)	1.62 (0.78–3.37)	1.33 (0.62–2.84)
Difficulty falling asleep (yes)	% (*n*)	Unadjusted OR (95% CI)	Adjusted^b^ OR (95% CI)
M-ABC test score ≤ 95th percentile	16.2 (31)	1.0	1.00
M-ABC test score > 95th percentile	25.6 (10)	1.78 (0.79–4.02)	1.50 (0.65–3.49)
Awakenings during the night (yes)	% (*n*)	Unadjusted OR (95% CI)	Adjusted^b^ OR (95% CI)
M-ABC test score ≤ 95th percentile	18.8 (36)	1.00	1.00
M-ABC test score > 95th percentile	25.6 (10)	1.49 (0.66–3.32)	1.15 (0.49–2.69)
Wake up unusually early (yes)	% (*n*)	Unadjusted OR (95% CI)	Adjusted^b^ OR (95% CI)
M-ABC test score ≤ 95th percentile	5.2 (10)	1.0	
M-ABC test score ≤ 95th percentile	10.3 (4)	2.07 (0.61–6.97)	^d^

OR, odds ratio, CI, confidence interval.

^a^ Gestational age <28 weeks or birth weight <1,000 g.

^b^ Adjusted for SGA and maternal education (dichotomized as less than a 3-year college education or not).

^c^ Movement Assessment Battery for Children.

^d^ Analysis not conducted due to small sample sizes.

### Sleep problems and NDD

The prevalence of all four types of sleep problems tended to increase with increasing severity of NDD, but the OR was only statistically significant when children in NDD 2 and 3 were compared to children without NDD (NDD 0) and only for general sleep problems, night awakenings and early morning awakenings. The OR was significant both in the unadjusted and adjusted regression analyses (Table 5).

**Table 5:** Parent-reported sleep problems at two to five years in children born extremely preterm^a^ in Norway in 1999 and 2000, according to the degree of neurodevelopmental disabilities^b^ (NDD) at five years. (*n* = 253).

General sleep problems (yes)	% (*n*)	Unadjusted OR (95% CI)	Adjusted^c^ OR (95% CI)
No NDD	20.5 (24)	1.00	1.00
NDD 1	30.0 (30)	1.66 (0.89–3.09)	1.61 (0.86–3.03)
NDD 2 and 3	50.0 (18)	3.88 (1.75–8.56)	3.87 (1.72–8.72)
Difficulty falling asleep (yes)	% (*n*)	Unadjusted OR (95% CI)	Adjusted OR (95% CI)
No NDD	13.7 (16)	1.00	1.00
NDD 1	20.0 (20)	1.58 (0.77–3.24)	1.53 (0.74–3.17)
NDD 2 and 3	25.0 (9)	2.10 (0.84–5.28)	2.01 (0.79–5.12)
Awakenings during the night (yes)	% (*n*)	Unadjusted OR (95% CI)	Adjusted OR (95% CI)
No NDD	13.7 (16)	1.00	1.00
NDD 1	24.0 (24)	1.99 (0.99–4.01)	1.92 (0.94–3.91)
NDD 2 and 3	41.7 (15)	4.51 (1.93–10.52)	4.77 (1.99–11.44)
Wake up unusually early (yes)	% (*n*)	Unadjusted OR (95% CI)	Adjusted OR (95% CI)
No NDD	2.6 (3)	1.00	1.00
NDD 1	6.0 (6)	2.43 (0.59–9.96)	2.34 (0.57–9.64)
NDD 2 and 3	16.7(6)	7.6 (1.79–32.18)	7.89 (1.83–33.93)

OR, odds ratio, CI, confidence interval.

^a^ Gestational age <28 weeks or birth weight <1,000 g.

^b^ Degree of neurodevelopmental disabilities (NDD): no NDD: No identified disability was defined as no cerebral palsy, full IQ (FIQ) 85 or higher, M-ABC score less than or equal to the 95th percentile, and normal vision and hearing. NDD 1: Minor disability was defined as cerebral palsy class 1, FIQ 1–2 SDs below mean (i.e., 70–84), an M-ABC score higher than the 95th percentile, squint/refractive error, or mild hearing loss. NDD 2: Moderate disability was defined as cerebral palsy class 2–3, FIQ 2–3 SDs below mean, severe visual impairment, or need of hearing aid. NDD 3: Severe disability was defined as 1 or more of the following: cerebral palsy class 4–5 on the Gross Motor Function Classification System for Cerebral Palsy, FIQ more than 3SDs below the reference mean value of 100, legal blindness, or complete deafness.

^c^ Adjusted for SGA and maternal education (dichotomized as less than 3-year college education or not).

## Discussion

In this national cohort, EPT children with moderate and severe NDD had a four to five-fold increased risk of having general sleep problems, nighttime awakenings, and early morning awakenings as compared to children without NDD at age two to five years. Low or borderline cognitive function as a separate variable was associated with a two to three-fold increased risk of the same sleep outcomes, while low motor function alone was not associated with sleep problems.

The prevalence of parent-reported general sleep problems was 28.5%, which is higher than the prevalence reported for unselected preschool children in a Norwegian study (19.2%) (43) and in an American study (10.8%) (44).

In the Norwegian study, parent interviews were conducted to assess various sleep problems, leading to the creation of a composite category for “any sleep disorder”, which included insomnia, hypersomnia, nightmare disorder, and sleepwalking (43). The study included all children born in two specific birth years within a geographical area, with the only exclusion criterion being children whose parents lacked sufficient language skills to complete the questionnaire. The American study is similar to ours in how sleep problems were assessed; parents were asked whether their children had a sleep problem, not through a questionnaire as in our study, but as part of an interview (44). This study was based on a national poll of parents of preschool children, selected through a targeted random sample.

Our findings align with previous research (6) and earlier studies in the same cohort (28). However, among the children in our cohort with no NDD, the prevalence of 20.5% was within range of the 19.2% reported for the unselected Norwegian population suggesting that extreme prematurity *per se* may not be a significant risk factor for sleep problems at two to five years of age. It is also important to note the potential uncertainty arising from the different methods and definitions used to classify sleep problems.

There are few studies on preschoolers born preterm that allow for direct comparison with our current findings. Our study identified a link between reduced cognitive abilities and early morning awakenings in EPT children. We have identified two studies that report similar findings (26, 27). Ando et al. (26) and Schwichtenberg et al. (27) found that increased variation in daily wake time and increased daily variation in sleep/activity cycles are associated with lower cognitive function in 1.5–2-year-old prematurely born children. We speculate that early morning awakenings and variations in wake-up time and sleep/activity cycles may result from the impact of premature birth on brain development, reflecting an underlying pattern that affects both sleep and cognitive development.

We also identified an association between early morning awakenings and moderate to severe NDD (NDD 2 and 3), which is consistent with our previous study of the same cohort at 11 years of age (28). Other studies of preterm adolescents and adults have also reported an increased prevalence of early morning awakenings (11, 12), but they have not examined the association between sleep and neurodevelopmental factors. The tendency of early morning awakenings among children born preterm may result from various mechanisms, including irregular sleep cycles, early chronotype (45, 46), and environmental influences (47). However, our finding of an association between decreasing FIQ and early morning awakenings likely indicates that the problem may be related to underlying neurological disturbances caused by preterm birth.

We found that borderline or low cognitive function was associated with increased nighttime awakenings, which differs from the findings of Ando et al., who reported no such association (26). However, their study differed markedly in that the children were born at higher GA.

Our finding that general sleep problems were associated with borderline or low cognitive function is comparable with two other studies: A study of 6–9-year-old children born at less than 32 weeks' GA where poor nighttime sleep quality was associated with poor verbal working memory (25) and a study of 6–10-year-old children where children born before 32 weeks' GA more often experienced nighttime awakenings than children born at term (20). Opposite to our findings, they found no association between nighttime awakenings and IQ (20), but the studies may not be comparable because their IQ levels were higher than in our children.

Nighttime awakenings in our two to five-year-old children born EPT may indicate developmental disruptions in the brain and altered development of the sleep-wake cycle. It is also possible that nighttime awakenings cause disruptions in sleep and thereby affect brain development, including cognitive abilities and daily cognitive functioning (15).

We suggest that the association between borderline and low IQ and sleep problems in our cohort of EPT children is clinically significant, as it can have profound implications for both immediate and long-term outcomes. While this association does not necessarily imply causality or etiology, it supports the hypothesis of shared root cause (13). It is also clinically significant in that IQ may be an indicator of neurological development in relation to sleep and, furthermore, that IQ remains relatively stable from early childhood to adulthood (48, 49), especially for those with low IQ (49). This may suggest that the sleep pattern of EPT children with borderline or low IQ may be stable over time and potentially add to their cognitive difficulties. However, this raises questions for further research, as sleep problems can potentially exacerbate the link between lower cognitive function and lower achievement (50).

Future research is necessary to further explore the mechanisms linking low cognitive scores and sleep problems in children born preterm. Longitudinal studies that track cognitive development and sleep patterns over time, along with interventions aimed at improving sleep quality and assessing their impact on cognitive outcomes, are essential. Furthermore, incorporating neurophysiological assessments may help reveal underlying brain changes that contribute to these relationships.

In general, addressing sleep issues in children is important, not only for ensuring optimal development (15), but also for their overall health and quality of life (51). Early identification and treatment of sleep problems in preschool children is crucial, as this period is significant for cognitive and social-emotional development (52). For EPT children, the association between sleep problems and neurodevelopmental outcomes identified in our study at this age is important for developing tailored follow-up interventions, which may ultimately support better developmental outcomes and enhance their overall quality of life.

## Strengths and limitations

The strengths of this study include a national population-based sample of EPT children, a prospective design, a high participation rate, and the use of validated instruments for assessing cognitive and motor function. The risk of selection bias was low because of extensive knowledge of non-participants.

Weaknesses include the lack of validated questionnaires assessing children's sleep problems, as well as the absence of a healthy reference group born at term. Furthermore, previous studies have shown that children born preterm are at risk of sleep-related breathing disorders (53–55), which are known causes of nighttime awakenings (56). Sleep-related breathing problems have also been shown to impact cognitive abilities (57). Unfortunately, we did not assess such sleep-related breathing problems in this study.

Weaknesses also include the fact that the included extremely premature (EPT) children were born in 1999 and 2000, which may raise concerns about the relevance of the findings for current populations. The survival rate and neonatal major morbidity in our cohort are comparable to today's survivors within the defined gestational age span of 24–27 weeks, both in Norway and in other high-income countries (58). Stensvold et al. report that no significant improvement in survival was found among viable infants in the 2013–2014 cohort compared to our cohort from 1999 to 2000. They found only small changes in the occurrence of major morbidity and no changes in mortality between the two cohorts (58). Data from the Norwegian neonatal quality register indicate that major morbidity during the neonatal period for survivors with gestational age (GA) <28 weeks has remained stable from 2012 to 2023, alongside some changes in survival rates for those with low GA in recent years (59).

However, it is important to be aware that neurodevelopmental outcomes are linked not only to major morbidity or major brain lesions but also to minor brain injuries, such as diffuse white matter abnormalities and other subtle brain injuries (60–62). We have limited knowledge about whether the improvements in treatment in recent years have affected the prevalence of these minor injuries. When the findings from our study are to be compared with those from newer studies, this must be taken into consideration. Additionally, the development of cognitive assessment tools has provided more sensitive instruments to capture neurological changes in preterm populations (63, 64).

Our study is a cross-sectional study, and the absence of longitudinal data is also a limitation. It is also important to acknowledge that factors such as genetics and environment are crucial for neurodevelopment (65). One significant environmental factor that has received scrutiny is the increased screen time among children in recent decades, which may negatively impact early neurodevelopment and has been shown to contribute to adverse cognitive outcomes (66), including for vulnerable EPT children (67). Since our cohort was raised in the early 2000s, any changes in evolving environmental factors must be taken into account, as this may limit the comparability of our findings with those of newer studies.

Socioeconomic factors may affect risks of preterm birth (37), lower cognitive abilities (40), and sleep problems (38). Although socioeconomic status encompasses multiple dimensions, we adjusted for maternal educational level in the adjusted analyses. There was only a modest increase in the proportion of individuals with higher education in Norway from year 2000 to 2020 (68) and no severe changes in other relevant factors such as employment rates (69). This social stability underscores our belief that the findings from our cohort are relevant for extremely premature infants born today.

## Conclusions

This study shows that various sleep problems are common among EPT children aged two to five years, and that sleep problems increase with borderline and low cognitive function and neurodevelopmental disabilities. Whether these associations have causal relationships or may interact negatively is not settled, but such a possibility cannot be dismissed. We therefore suggest that sleep behavior needs to be addressed in the follow-up of children born preterm.

## Data Availability

The datasets presented in this article are not readily available because according to the approvals granted for this study by The Regional Committee on Medical Research Ethics and The Norwegian Data Inspectorate, the data files are to be stored properly and in line with the Norwegian Law of Privacy Protection. The data file is not made publically available as this might compromise the respondents' privacy, particularly as some of our participating centers are small and the number of extremely preterm births very limited. Moreover, the data file is currently used by other researchers in our group to prepare future research papers. A subset of the data file with anonymized data may be made available to interested researchers upon reasonable request to Thomas Halvorsen (thomas.halvorsen@helse-bergen.no) and providing permission from The Norwegian Data Inspectorate and the other members of our research group. Requests to access the datasets should be directed to thomas.halvorsen@helse-bergen.no.
